# Generation of Urine Cell-Derived Non-integrative Human iPSCs and iNSCs: A Step-by-Step Optimized Protocol

**DOI:** 10.3389/fnmol.2017.00348

**Published:** 2017-10-30

**Authors:** Lin Cheng, Qiannan Lei, Chen Yin, Hui-Yun Wang, Kangxin Jin, Mengqing Xiang

**Affiliations:** ^1^State Key Laboratory of Ophthalmology, Zhongshan Ophthalmic Center, Sun Yat-sen University, Guangzhou, China; ^2^State Key Laboratory of Oncology in South China, Sun Yat-Sen University Cancer Center, Guangzhou, China; ^3^Department of Pediatrics, Center for Advanced Biotechnology and Medicine, Rutgers University-Robert Wood Johnson Medical School, Piscataway, NJ, United States

**Keywords:** iPSC, iNSC, urine cell, reprogram, protocol

## Abstract

**Objective:** Establishing a practical procedure to generate induced pluripotent stem cells (iPSCs) and induced neural stem cells (iNSCs) from human urine cells (UCs). In this report, we optimized a non-integrative protocol to generate patient-specific iPSC and iNSC lines with high reprogramming efficiency.

**Methods:** UCs were electroporated with the pEP4-EO2S-ET2K and pEP4-M2L plasmids containing the *OCT4, SOX2, KLF4, SV40LT, c-MYC*, and *LIN28* genes, and then cultured with N2B27 medium plus four small molecule compounds (A83-01, PD0325901, Thiazovivin, and CHIR99021). When iPSC or iNSC clones emerged, the medium was replaced with mTeSR1 or neural growth medium. Morphological changes were seen at day 4–7. After day 10, the clones were picked up when the clone diameter exceeded 1 mm.

**Results:** iPSCs and iNSCs were successfully derived from UCs with up to 80 clones/well. These iPSCs and iNSCs showed typical hESC or NSC morphology and were self-renewable. The iPSCs had pluripotency to differentiate into the three germinal layers and displayed high levels of expression of pluripotency markers SOX2, NANOG, OCT4, SSEA-4, TRA-1-60, TRA-1-81, and alkaline phosphatase (AP). They maintained normal karyotype and had no transgene expression or genomic integration. The iNSCs were positive for NSC markers NESTIN, PAX6, SOX2, and OLIG2.

**Conclusion:** The optimized protocol is an easy and fast procedure to yield both iPSC and iNSC lines from a convenient source of human urine in a single experiment.

## Introduction

Recapitulating the developmental mechanism of organoids represents exciting areas of research that have opened new avenues for understanding organ development, inherited diseases and diseases related to aging and environment. The induced pluripotent stem cells (iPSCs) and induced neural stem cells (iNSCs) not only provide unlimited source of cells for disease modeling, drug test and screening, and studying the dynamic developmental processes of tissues, but also allow us to generate desired organoids for autologous transplantation and manipulate genes to better understand diseases.

A large number of somatic cells have been reprogrammed into iPSCs or iNSCs, including fibroblasts, keratinocytes, melanocytes, adipose cells, peripheral blood cells, periosteum membrane cells, hepatocytes, and amniocytes (Zhou et al., 2012). Urine cells (UCs), which are exfoliated renal system epithelial cells, are able to be collected under any circumstances except renal failure. Therefore, urine exfoliated cells provide us with a practical and unlimited source of human cells for reprogramming, and this non-invasive way of taking human cells would largely increase the patient's compliance. In addition, eliciting epithelial-to-mesenchymal transition is essential for somatic cells to become stem cells, and UCs, as renal epithelial cells, are easier to overcome this conversion due to their epithelial origin.

Hence, by using the UCs, we optimized a non-integrative method through introducing two episomal plasmid DNAs into the cells to generate iPSCs and iNSCs. Based on previously published methods (Okita et al., 2011; Yu et al., 2011; Xue et al., 2013; Li et al., 2016), we leveraged on defined culture medium to refine a standardized protocol for rapid generation of iPSCs and iNSCs. This protocol is feeder-free, integration-free, and highly efficient. The protocol increases the reprogramming efficiency by the use of 4 inhibitors (4i): ROCK inhibitor Thiazovivin, MEK inhibitor PD0325901, GSK-3α/GSK-3β inhibitor CHIR99021, and TGF-β/Activin/Nodal receptor inhibitor A83-01. The iPSCs and iNSCs reprogrammed from UCs develop at twice the speed of iPSCs and iNSCs generated from blood or skin cells.

UC-derived iPSCs and iNSCs are useful tools in cell-based therapies and tissue engineering. UC-derived iPSCs can be differentiated into tooth, bone, cartilage, fat, skeletal muscle, urothelium, and smooth muscle cells, etc. (Bharadwaj et al., 2013; Cai et al., 2013). Researchers have also established iPSCs from children's urine samples to study autism spectrum disorders (Baker, 2012). UC-derived iNSCs can generate functional neurons and survive up to 1 month after transplantation into the mouse brain (Wang et al., 2013). This may help to develop therapies for neurodegenerative diseases such as Alzheimer's and Huntington's. By taking advantage of patient-specific UCs, these feeder-free, serum-free, integration-free, and clinical grade iPSC and iNSC lines can provide unlimited source of cells for disease modeling, transplantation, or evaluation of toxicity and effects. In addition, iPSCs/iNSCs are ideal targets for gene manipulation to better understand the underlying mechanism of disease.

## Ethic statement

The study was approved by the Institutional Review Board of Sun Yat-sen University (2017KYPJ062) and conducted in accordance with the tenets of the Declaration of Helsinki. Written informed consent forms were obtained from patients after detailed explanation for the study protocol.

## Reprogramming procedures

### 1. Urine cell collection

The procedure for urine sample collection and isolation is detailed in Zhou et al. (2012). In brief, coat beforehand the 12-well plate with 0.1% gelatin for 1 h. Collect 100–300 ml urine with a 500 ml autoclaved bottle and transfer the samples to sterile 50 ml tubes. Centrifuge the tubes at 400 rcf for 10 min and keep ~1 ml sedimentation in the tubes. Resuspend the pellets gently and combine each ~1 ml urine together into a single 50 ml tube. Add 10 ml washing buffer, mix thoroughly, and centrifuge the samples at 200 rcf for 10 min. Remove the supernatant carefully and leave ~0.2 ml plus the pellet. Add 1 ml of primary medium to the pellets, resuspend the pellets and transfer them into a 12-well plate. This is considered as day 0 of the urine cell culture. Add 1 ml primary medium for the first 3 days without removing any medium. At day 4, remove most of the medium (~3 ml) and leave ~1 ml in the plate. Add 1 ml RE/MC medium. In the next few days, change half of the medium with RE/MC medium and keep the other half. Small UC colonies should appear from 3 to 5 days. Culture the UCs with RE/MC medium and split the cells 1:3 or 1:4 (depends on the growth of UCs) to a 6-well plate when it reaches 80-90% density. Healthy UCs should expand quickly. Alternatively, UCs can be frozen with UC freezing medium in liquid nitrogen for later use **(pause point)**.

**Figure 1:**
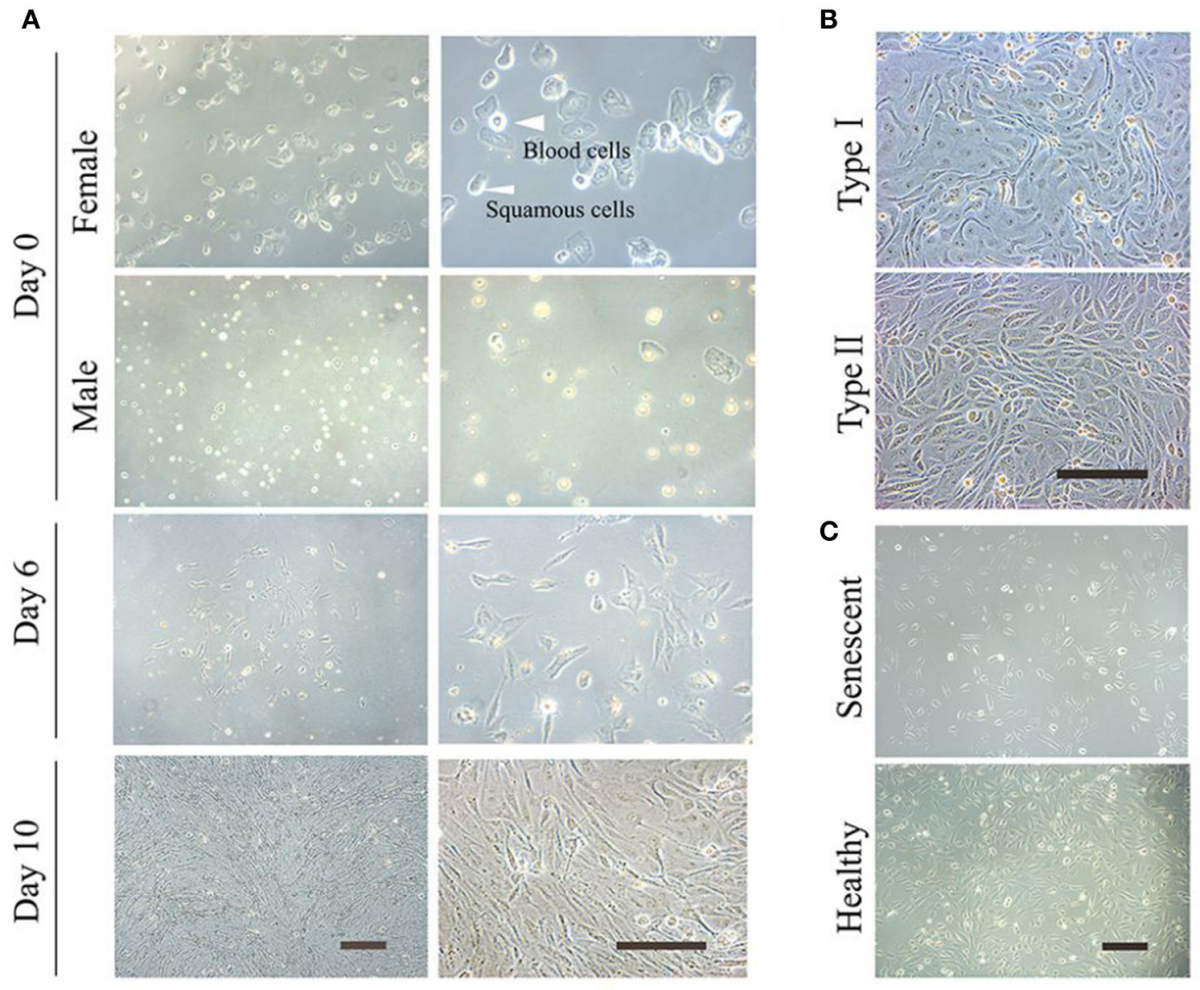
Culture of the exfoliated urine cells. **(A)** The urine cell culture from day 0 to day 10. Female urine samples contain squamous cells, and occasionally a few of blood cells. Male samples contain round-shaped sediments. After culture for about 3–4 days, small colonies of epithelium-like cells starts to appear (see day 6). After expansion (day 10), the cells become closely packed. **(B)** The two types of urine cells. Type I cells have smooth-edged contours and cobblestone-like shape. Type II cells are in slender form and more randomly arranged. **(C)** The comparison of senescent and healthy urine cells. All scale bars: 200 μm.

The protocol presented here reprograms UCs into iPSCs and iNSCs. It is important to start with healthy, non-senescent UCs (Figure 1).

### 2. Pre-nucleofection

#### Day−1, and −2

2.1 Plate human UCs in RE/MC medium on a 0.1% gelatin-coated 6-well plate. The cells can be either cryopreserved cells or freshly isolated UCs. Make sure that you have at least twice more of the cells needed for transfection because some cells may be lost during digestion. The ideal cell number is ~1 × 10^6^ cells/well, which is 10 times more than that (1–3 × 10^5^ cells/well) used in Wang et al. (2013).

Note: When preparing the plasmids for reprogramming, we recommend using the Qiagen® EndoFree Maxi Plasmid Extraction Kit. By excluding endotoxin and increasing DNA purity, it helps to increase the reprogramming efficiency. The suggested plasmid concentration is more than 1 μg/μl. Alternatively, you may use the manual chloroform extraction method to obtain high-quality plasmid DNA.

#### Day 0

2.2 Make sure that the UCs are healthy and reach 80-90% confluency at the time of transfection.
2.3 Three hours before electroporation, change the medium to RE/MC medium supplemented with 2 μM Thiazovivin. Treatment with ROCK inhibitor enables UCs to better tolerate electroporation.
2.4 Coat a new 6-well plate with 1% hESC-qualified matrigel (dilute with DMEM/F12, 1 ml/well, >1 h).
2.5 Aspirate the matrigel, add 3 ml RE/MC medium plus 2 μM Thiazovivin per well. Place the 6-well plate with medium in 37°C incubator until needed.
2.6 Prewarm HBSS, 0.25% Trypsin-EDTA, and trypsin inhibitor to 37°C.
2.7 Start up 4D-Nucleofection™ system and set experimental parameters. Select for the appropriate nucleofection program. We select “PrEC, human” in “cell type program.”

### 3. Nucleofection

3.1 Aspirate the medium from the plate wells. Wash the cells once with HBSS (2–3 ml).
3.2 To harvest cells, add 0.8 ml 0.25% Trypsin-EDTA to each well. Incubate the plate in 37°C/5% CO_2_ incubator until the cells are completely detached.
3.3 Neutralize trypsinization reaction with trypsin inhibitor.
3.4 Transfer the dislodged cells to a 15 ml conical tube.
3.5 Centrifuge the cells at 200 rcf for 5 min. Aspirate the supernatant and resuspend the cells in 3 ml PBS or HBSS. Count the cells with a hemocytometer. Divide the cells into different microcentrifuge tubes by filling appropriate number of cells (1.5 × 10^6^ cells/tube).
3.6 Centrifuge the cells at 400 rcf for 5 min. Aspirate most of the supernatant and remove the remaining supernatant with a 200 μl pipette. Note: This step is critical because the divalent ions reduces the nucleofection effect.
3.7 Prepare the 100 μl nucleofection reagent in a microcentrifuge tube (82 μl nucleofection solution and 18 μl supplement for the 100 μl system). Add two oriP/EBNA1-based pCEP4 episomal vectors containing the *OCT4, SOX2, KLF4, SV40LT, c-MYC* and *LIN28* genes, pEP4-EO2S-ET2K (6 μg) and pEP4-M2L (4 μg) (Yu et al., 2009), to the tube with nucleofection reagent. Add the control plasmid pmaxGFP vector (2 μg, 1 μg/μl, 2 μl) provided by the kit to the control tube. Do not vortex the plasmids vigorously, as it damages the plasmid circular structure. Mix by tapping or pipette up and down 5×.
3.8 Resuspend the cell pellet carefully at room temperature in the 100 μl 4D-Nucleofection^TM^ reagent/plasmid mix prepared above. The final concentration is 1.5 × 10^6^ cells/100 μl.
3.9 Transfer each episomal iPSC reprogramming mixture to the sterile nucleofection vessel. If the solution is >100 μl, discard it as the electroporation capacity is 100 μl. Note: Maximally over 10% of final sample volume is allowed.
3.10 Electroporate the cells with the cell type program set above.
3.11 Plate all electroporated cells from one reaction into one well of the matrigel-coated 6-well plate with RE/MC medium supplemented with 2 μM Thiazovivin set up beforehand.
3.12 Incubate the plate in 37°C/5% CO_2_ incubator overnight. This is considered day 0 of nucleofection.

### 4. Post-nucleofection

4.1 Check GFP expression at around 15 h later to assess the nucleofection efficiency.
4.2 Replace the medium with fresh RE/MC medium supplemented with A83-01 (0.5 μM), PD0325901 (0.5 μM), Thiazovivin (0.5 μM), and CHIR99021 (3 μM) (4i) on day 2 or day 3. Change this medium every other day but for no more than two times. If the majority of cells are dead, change the medium as early as possible. This ensures that the surviving cells proceed to the reprogramming step at earlier times. Monitor the morphology change of the UCs under a phase-contrast microscope daily. Typically, the cells exhibit morphological change as early as day 3. The cells usually change from a long-strip shape to a round shape with a large nucleus. The nucleo-cytoplasmic ratio is significantly higher than that of UCs, and most of the cells become compact and dense, and closely packed to each other.

### 5. iPSC or iNSC fate decision

5.1 Switch to N2B27 medium supplemented with 4i (the concentrations are the same as above, see Table 1) when confluence is more than 80%. Note: The concentration and treating period of 4i compounds for the cells are important. It determines iPSC, iNSC or iPSC/iNSC generation. If 4i compounds are in higher concentrations than recommended or the 4i-containing medium is changed more often, the iPSCs appear earlier; if less 4i compounds are added, iPSCs emerge later. Observe the morphological change under a phase-contrast microscope daily, using the 4 × or 5 × objectives. Change the medium every other day.
5.2 For iPSC reprogramming, if the peripheral part of the clone starts to upheave, and the clone starts to grow layer by layer up, it is the time to change for mTeSR1 medium (day 10–12) to let the clone grow big enough for picking.
5.3 For iNSC reprogramming, 4i treatment could exceed 15 days post-electroporation (day 12–15 and later). When the clone upheaves, continuous culture of the cells with N2B27 medium plus 4i results in iNSCs. Observe the morphological change and determine how long you want to treat the cells (day 15 or longer). The number of treating days is subject to change according to each cell condition. Therefore, monitoring cell morphological change is critical to determine the period of treatment for the generation of proper cell type. Examples of specific morphological changes of iPSCs and iNSCs are shown in **Figure 5A**.

**Table 1:** Stock and working concentrations of 4i compounds for regrogramming.

**Compounds**	**CAS#**	**Molecular formula**	**Molecular weight**	**Stock fold**	**Stock concentration (mM)**	**Working concentration (μM)**
A83-01	909910-43-6	C_25_H_19_N_5_S	421.52	10,000 ×	5	0.5
PD0325901	391210-10-9	C_16_H_14_F_3_IN_2_O_4_	482.19	10,000 ×	5	0.5
Thiazovivin	2656-71-8	C_15_H_13_N_5_OS	311.36162	10,000 ×	5	0.5
CHIR99021	252917-06-9	C_22_H_18_CI_2_N_8_	465.34	1,000 ×	3	3

*Higher concentrations of chemical factors in this protocol may result in toxicity to the reprogrammed cells. CHIR99021 and A83-01 are fatal if swallowed, and can cause skin irritation, serious eye irritation and respiratory irritation. Avoid breathing these two compounds and contact with skin and eyes. PD0325901 is toxic if swallowed, and may cause damage to organs through prolonged or repeated exposure and long lasting harmful effects to aquatic life. Avoid releasing PD0325901 to the environment. Thiazovivin is not a hazardous substance or mixture. When dealing with the 4i compounds, do it in a well-ventilated area. Wear protective gloves, eye protection, and face protection*.

### 6. Clone pickup

6.1 When the clone diameter exceeds 1 mm, the clones can be picked up for further expansion.
6.2 Transfer the dislodged iPSC/iNSC colonies to a 24-well matrigel-coated plate mechanically. If the cells are picked up too early or the cells are too few to fill the plate, the cells can easily differentiate. For further expansion, iPSCs are dissociated to small clumps by dispase and cultured with mTeSR1 on matrigel-coated plate. iNSCs are dissociated to small clusters with accutase and cultured in neural growth medium on plates coated with 1% matrigel. When expanded for 1–2 passages, iPSCs and iNSCs can be cryopreserved for further expansion or characterization **(pause point)**.

## Anticipated results

UCs are at high risk of contamination if not handled appropriately. We recommend cleaning the genitalia with hygienic towelette and collecting the continuous flow of midstream urine. Do not touch the bottleneck and the inside of the cap. If the cells show mild contamination, wash them thrice with newly prepared washing buffer and add primocin™ to the RE/MC medium. Primocin™ is an antibiotic formulation designed to protect primary cell lines from cell culture contaminations. After treatment, the mild contamination can be eliminated. If not, discard the cells. To increase the yield, drinking abundant water (>500 ml) in a short period 2 h beforehand and extracting the UCs freshly can significantly increase the cells. If no UC colonies are observed, collect more volume and combine several collections to one well. Do not use the first micturition urine as the viability of UCs in it is low. If there are too many sediments such as squamous cells in the culture plates, remove the majority of squamous cells to another 0.1% gelatin-coated 12-well plate after culture day 4. Culture them with RE/MC medium. If no UC colonies are seen, discard them.

**Figure 2:**
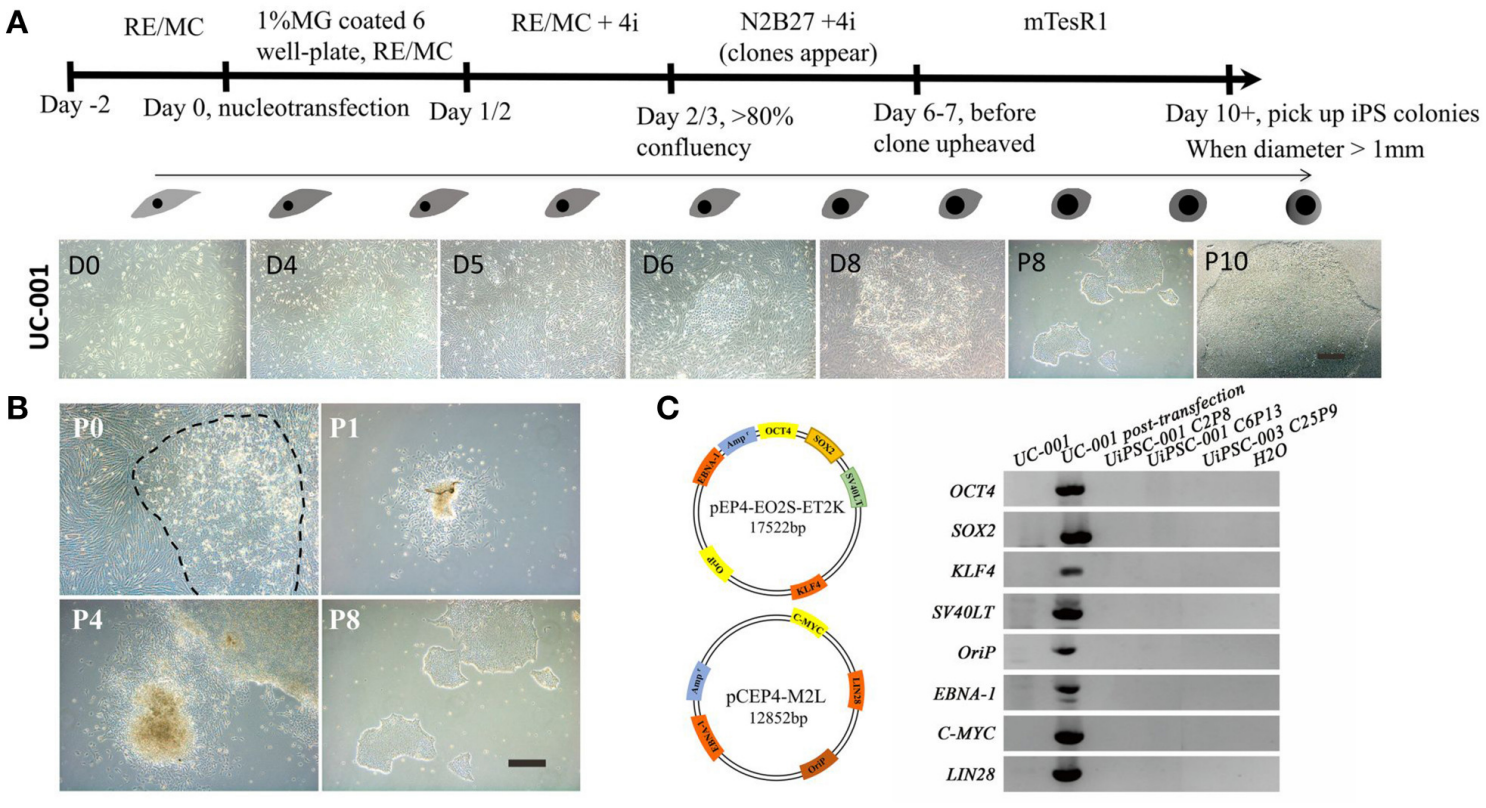
Reprogramming of non-integrative iPSCs from UCs. **(A)** The workflow of UC-derived iPSC (UiPSC) reprogramming. The figure illustrates the reprogramming procedures (upper) and the shape change from UCs to iPSCs from day(D)0 to passage(P)10 (lower). Scale bar: 200 μm. **(B)** Example of successful establishment of an iPSC line. Scale bar: 200 μm. **(C)** Non-integration of UC-derived iPSCs. The left plasmid cartoons illustrate the two plasmid backbones and the inserted genes. The right image shows a PCR test for non-integration of these plasmids in established iPSC lines. By using UCs and H_2_O as negative controls, and UCs at 3 days post-transfection as a positive control, PCR analysis showed that UiPSC lines had no integration of episomal plasmids.

**Figure 3:**
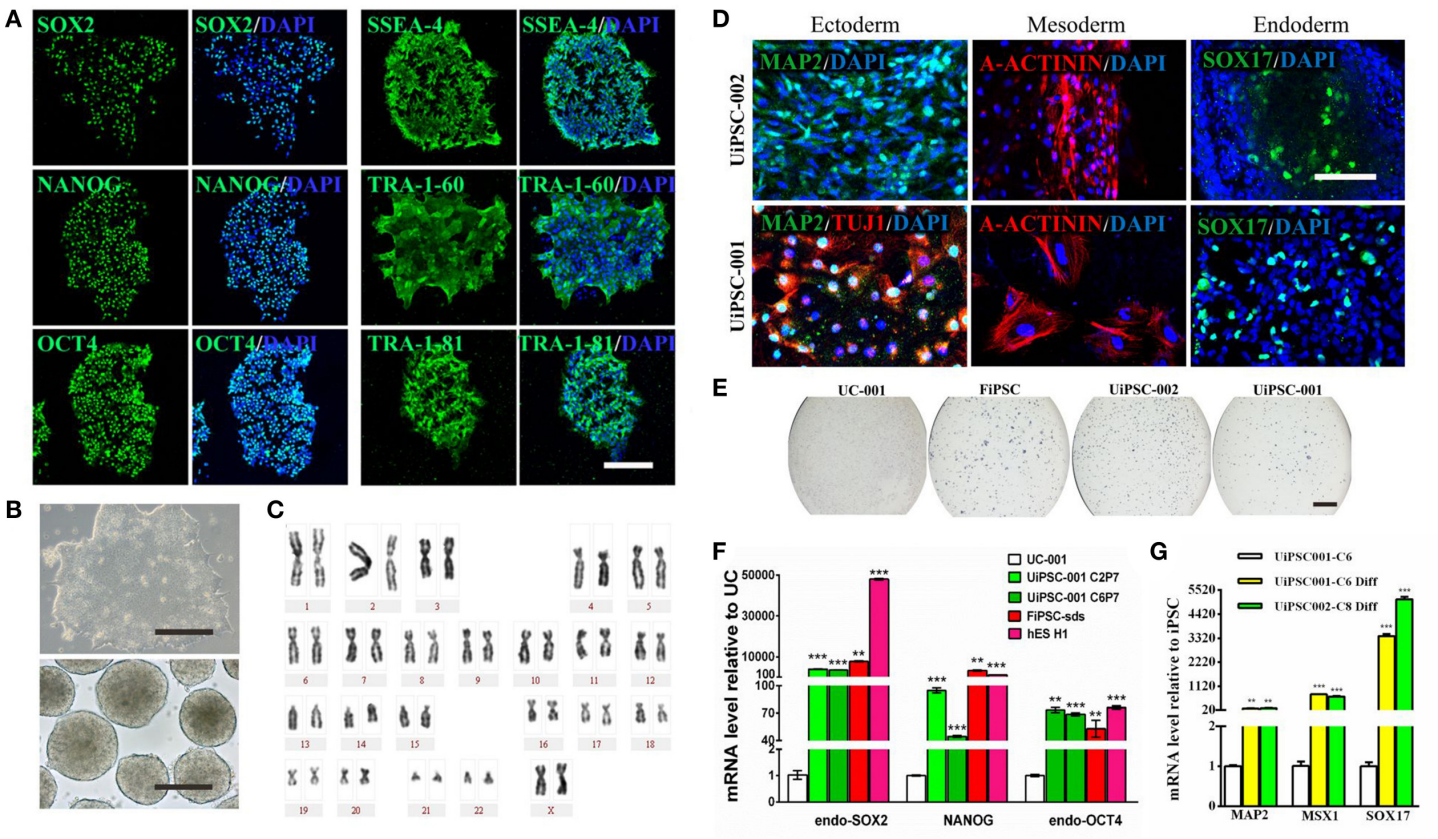
Characteristics of the UiPSCs. **(A)** Immunofluorescent labeling for pluripotency markers SOX2, NANOG, OCT4, SSEA-4, TRA-1-60, and TRI-1-81. **(B)** The morphology of UiPSCs cultured in mTeSR1 medium (upper) and differentiation medium (lower). **(C)** G-band analysis of an UiPSC shows normal karyotype. **(D)** Immunofluorescent labeling for three germinal layer markers following UiPSC differentiation. **(E)** AP staining of UiPSCs, with UCs and fibroblast-derived iPSCs (FiPSCs) as negative and positive controls, respectively. **(F)** qRT-PCR assay for expression of endogenous human pluripotency genes in two UiPSC lines, with UCs as negative control, and FiPSCs and human ES line H1 as positive controls. **(G)** qRT-PCR analysis for expression of three germ layer marker genes in UiPSCs after differentiation. Scale bar: A, B, D, 200 μm; E, 2 mm.

**Figure 4:**
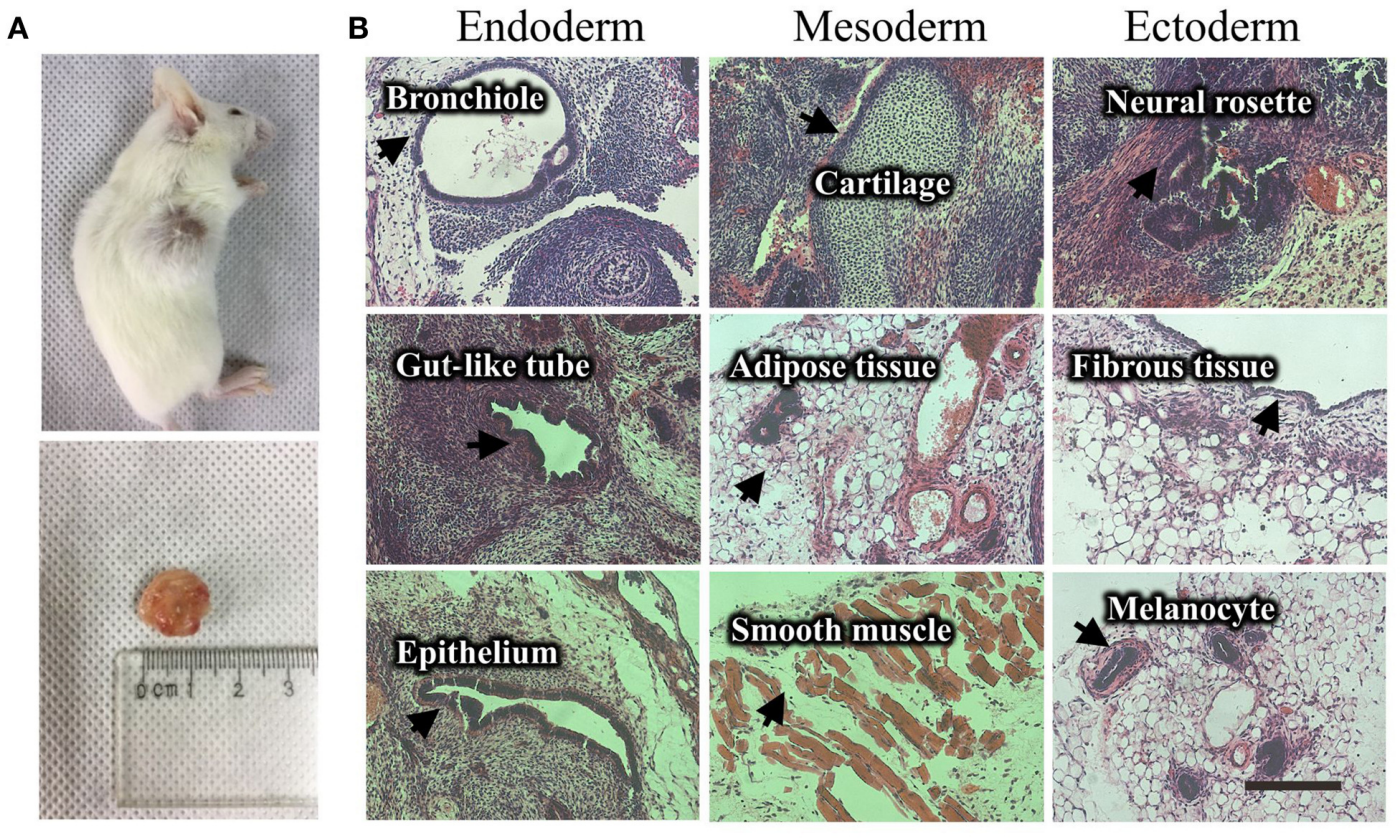
Teratoma formation of a UiPSC line. **(A)** UiPSC injection into immune-deficient mice (NOD-SCID) led to teratoma formation. **(B)** Hematoxylin-eosin (HE)-staining revealed that the generated teratomas contain the three germinal layer tissues. Scale bar: 200 μm.

**Figure 5:**
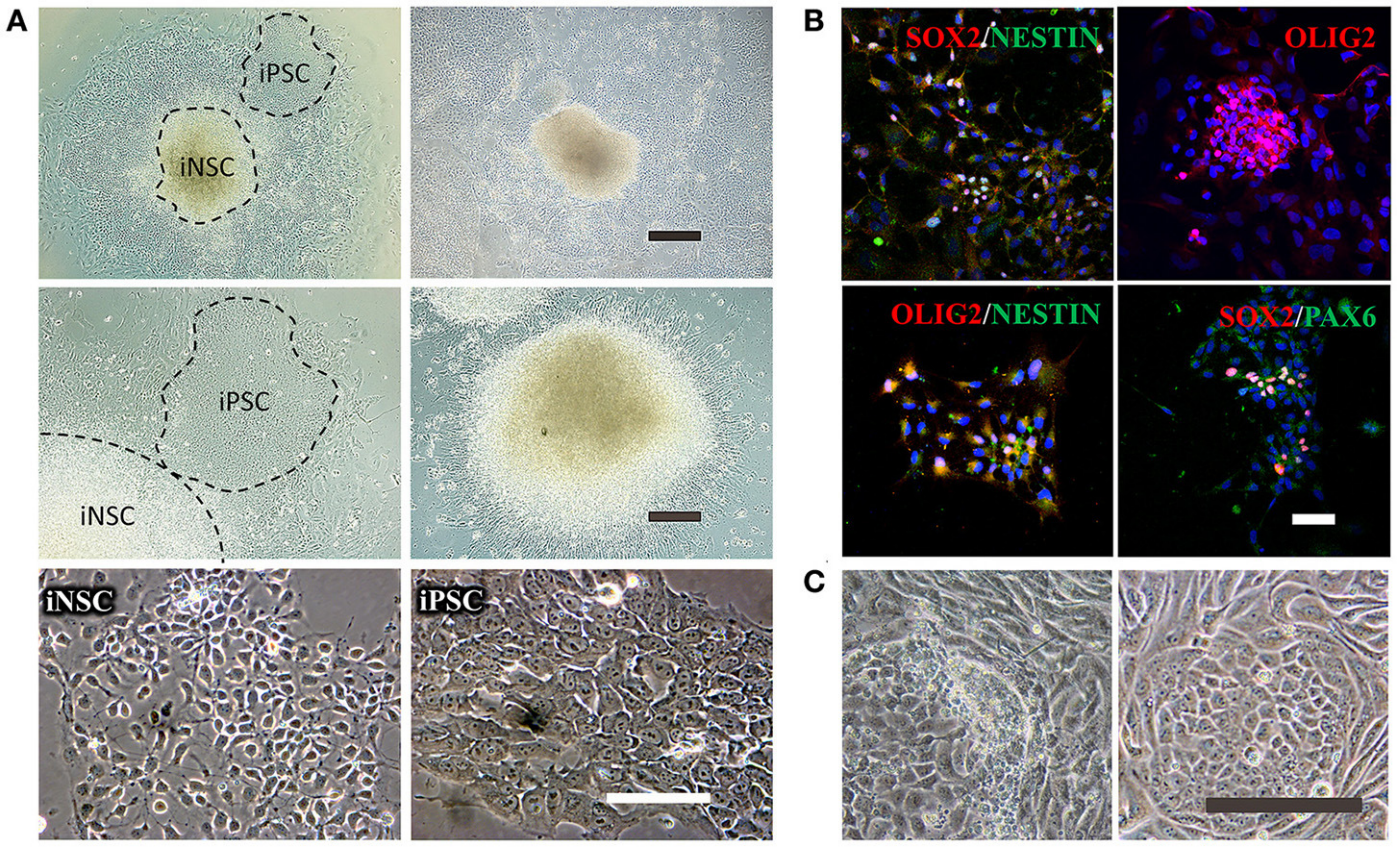
Reprogramming of iNSCs from UCs and comparison of a successful iPSC reprogramming and an unsuccessful iPSC reprogramming. **(A)** The morphology of iNSCs derived from UCs. The upper panel shows the UC-derived iPSCs and iNSCs (UiNSCs) reprogrammed at the same time and the UiNSCs after single-colony replating. Scale bar: 100 μm. The middle panel shows higher-magnification images of iPSCs and iNSCs and the neural rosette of iNSCs. Scale bar: 200 μm. The lower panel shows the morphology comparison between iNSCs and iPSCs. Scale bar: 100 μm. **(B)** Immunofluorescent staining of iNSC markers SOX2, NESTIN, OLIG2, and PAX6, with nuclear DAPI counter-staining. Scale bar: 100 μm. **(C)** Compared with a successful iPSC reprogramming in the right panel, the left image shows that the UCs underwent shape transformation but were not fully reprogrammed. Some cells had semi-shape transformation (not round) and the clone cells were loosely attached to each other. Scale bar: 200 μm.

This non-integrative optimized protocol is a simple, fast and efficient procedure to generate patient-specific iPSCs and iNSCs (Figures 2, 3). It results in the generation of iPSCs at around 10 days and iNSCs at around 12–15 days. The iPSCs showed typical hESC morphology and were self-renewable. They maintained normal karyotype (one line at P8 and two lines at P13) and were AP-positive. PCR assay confirmed that these iPSC colonies had no transgene expression or genomic integration (Figure 2C). Occasionally, some lines may have integration of plasmid genes (2/13), and usually these integration becomes lost after passage 15; otherwise, these lines should be discarded for clinical use purpose. qRT-PCR and immunofluorescence staining both confirmed that iPSCs displayed high levels of expression of pluripotency markers SOX2, NANOG, OCT4, SSEA-4, TRA-1-60, and TRA-1-81 (Figures 3A,F). The iPSCs showed excellent differentiation potential to form three germinal layers both *in vitro* and *in vivo* (Figures 3D,G, 4, 5). The iNSCs showed typical NSC morphology, and were positive for NSC markers NESTIN, PAX6, SOX2 and OLIG2 (Figure 5B). After being picked up, the iPSCs and iNSCs are cultured in mTeSR1 and neural growth medium on the plate coated with 1% matrigel, respectively. These protocols can be easily found elsewhere (Okabe et al., 1996; Wang et al., 2013; Chatterjee et al., 2016).

To yield a high reprogramming efficiency from UCs, we recommend the Amaxa® 4D-Nucleofector™ system together with the Amaxa® Basic Nucleofector™ Kit for nucleofection. If there is no access to this specific equipment, Neon® Transfection System working together with Neon® Transfection System 100 μl Kit (Thermo Fisher, MPK5000S) is also recommended. Alternatively, lipofection using the Lipofectamine 3000 (Thermo Fisher, 1882752) or X-tremeGENETM 9 DNA Transfection Reagent (Roche, 6365787001) can also introduce the plasmid DNAs into cells. The use of non-senescent UCs and highly purified endotoxin-free plasmids can increase the reprogramming efficiency and result in more iPSC or iNSC colonies. By contrast, senescent or contaminated UCs (e.g., mycoplasma containination) do not undergo shape transformation or only undergo semi-shape transformation, and fail to generate iPSCs or iNSCs. We recommend 1 × 10^6^ UCs/well in 6-well plate as a standard protocol. If less cell electroporation is unavoidable or the cells have tendency of senescence, electroporation early is better than later. In the case with less cells, use the 12-well plate or 24-well plate and scale the system accordingly. The reprogram efficiency may be sacrificed, but iPSCs/iNSCs can be established. Moreover, there is heterogeneity of reprogramming efficiency for UCs. For instance, UCs of different collections from the same donor may have different efficiency. This protocol, however, is highly reproducible and yields good-quality iPSCs and iNSCs every time.

## Pitfalls or artifacts

Pitfall 1: UCs are not attached to the bottom. The floating cells are very likely to be squamous cells or other sediments. The UC colonies are attached to the bottom at day 3–5.
Pitfall 2: UCs grow slowly. It is possible that UCs are senescent or have mycoplasma contamination. Senescent UCs have an appearance of expanded cell body with tentacles and blurring contours.
Pitfall 3: Weak or no GFP fluorescence of UCs after electroporation. Check if the electroporation reagents are expired, and make sure that the nucleofection vessel placed in the right direction. After electroporation, the equipment should show a “+” symbol.
Pitfall 4: Most UCs die the first day after electroporation. The electroporation program is wrong or there is no pre-treatment of ROCK inhibitor. The cells should be cultured with ROCK inhibitor on the first day after electroporation.
Pitfall 5: No morphology change of UCs or semi-shape transformation of UCs. The plasmids are wrong or the UCs are senescent if no morphology change is seen. Use the right plasmids and reprogram at an earlier passage. Semi-shape change is likely due to contamination (Figure 5C). Check if there is mycoplasma contamination.
Pitfall 6: UCs undergo shape change but do not show typical ESC-like morphology. N2B27 medium or 4i compound preparation problem or the cells are contaminated.
Pitfall 7: Failure in iPSC or iNSC expansion. If the iPSCs or iNSCs are differentiated after picking up, there may be a matrigel problem or the cells are picked up too early. The plate coating should be invisible. If some visible filaments can be seen after coating, it indicates the matrigel is denatured. The temperature should be between 0 and 4°C when handling matrigel. Pick up the colonies when the diameter is more than 1 mm.

## Materials

### Reagents

- Human urine
- Autoclaved bottles
- PBS (Thermo Fisher, 10010023)
- Penicillin/streptomycin solution (100×, Gibco, 15140-122)
- Antibiotic-antimycotic (100× Thermo Fisher, 15240-096)
- REGM SingleQuot kit supplement & growth factors (Lonza, CC- 4127)
- REGM (Renal Cell Growth Medium) BulletKit (Lonza, CC-3190)
- Gelatin (Sigma, G2500)
- 0.25% Trypsin-EDTA (Gibco, 25200-056)
- Primocin (InvivoGen, ant-pm-2)
- GlutaMAX™ Supplement (Thermo Fisher, 35050061)
- HBSS (Gibco, 14175-095)
- Trypsin inhibitor (Thermo Fisher, R-007-100)
- pEP4-EO2S-ET2K (Addgene, 20927), which contains *OCT4, SOX2, KLF4*, and *SV40LT* genes
- pEP4-M2L (Addgene, 20926) which contains *c-MYC, LIN28*
- EndoFree Maxi Plasmid extraction kit (Qiagen, 12362)
- DMEM high glucose (Hyclone, SH30243.01)
- hESC-qualified matrigel matrix (Corning, 354277)
- DMEM/F-12, GlutaMAX™ (Gibco, 10565-018)
- DMEM/F-12 (Gibco, 11320-082)
- β-mercaptoethanol 100X for ES (Millipore, ES-007-E)
- NEAA 100X (Gibco, 11140-050)
- Dispase (Stemcell technologies, 7923)
- N2 supplement (Gibco, 17502-048)
- B27 serum free supplement (Thermo Fisher, 17504-044)
- P1 Primary Cell 4D-Nucleofector™ X Kit L (12 RCT) (Lonza, V4XP-1012, 100 μl system)
- Thiazovivin (Sigma, SML1045)
- CHIR99021 (Sigma, SML1046)
- A83-01 (Sigma, SML0788)
- PD0325901 (Sigma, PZ0162)
- mTeSR1 (Stemcell technologies, 05850)
- bFGF (Peprotech, 100-18B)
- EGF (Peprotech, AF-100-15)
- PDGF-AB (Peprotech, 100-00AB)
- FBS (fetal bovine serum, Gibco, 10270-106)

### Equipment and supply

- Amaxa® 4D-Nucleofector™ X unit (Lonza, AAF-1002X)
- Centrifuge (Eppendorf, 5810R and 5424)
- Biological Safety Cabinets (Thermo Fisher, MSC-Advantage)
- CO_2_ incubator (Thermo Fisher, Heracell 150i)
- Phase-contrast microscope (Nikon, eclipse TS100)
- Bright-line hemocytometer (Qiujing Scientific, XB-K-25)
- 15 ml polypropylene conical tube (Falcon, 352096)
- 24-well plate (Corning, 3524)
- 6-well plate (Corning, 3516)
- 12-well plate (Corning, 3513)
- Microcentrifuge tube (Axygen, MCT-150-G)

### Reagent setup

- Washing buffer: 1× antibiotic-antimycotic in PBS
- Primary medium: 500 ml DMEM/F12 supplemented with 10% FBS, 1× antibiotic-antimycotic, REGM SingleQuot supplement & growth factors
- RE/MC medium: half REGM medium (Lonza, CC-3190) and half MC medium (DMEM/high glucose supplemented with 10% FBS, 1% GlutaMAX, 1% NEAA, 1× penicillin/streptomycin, 5 ng/ml bFGF, 5 ng/ml PDGF-AB, and 5 ng/ml EGF)
- UC freezing medium: RE/MC medium supplemented with 10% DMSO and 10% FBS
- N2B27 medium: DMEM/F12, GlutaMAX supplemented with 1× N2, 1× B27, 0.1 mM NEAA, 0.1 mM β-mercaptoethanol, and 100 ng/ml bFGF
- 4i: The compound stock and working concentrations are listed in Table 1
- Neural growth medium:1:1 of DMEM/F12 supplemented with 1% N2 and neurobasal medium supplemented with 2% B27, 20 ng/ml bFGF and 20 ng/ml EGF

## Author contributions

LC, KJ, and MX conceived and optimized the protocol. LC and QL performed lab experiments. CY and HYW helped with teratoma experiments. LC analyzed the data and wrote the paper. All the above authors contributed to the final paper.

### Conflict of interest statement

The authors declare that the research was conducted in the absence of any commercial or financial relationships that could be construed as a potential conflict of interest.
